# Evidence for Regulated Interleukin-4 Expression in Chondrocyte-Scaffolds under *In Vitro* Inflammatory Conditions

**DOI:** 10.1371/journal.pone.0025749

**Published:** 2011-10-03

**Authors:** Muhammad Farooq Rai, Thomas Graeve, Sven Twardziok, Michael F. G. Schmidt

**Affiliations:** 1 Institute of Immunology and Molecular Biology, Department of Veterinary Medicine, Freie Universität Berlin, Berlin, Germany; 2 Amedrix GmbH, Esslingen, Germany; 3 Institute of Molecular Biology and Bioinformatics, Charite University of Medicine, Benjamin Franklin Campus, Berlin, Germany; The University of Hong Kong, Hong Kong

## Abstract

**Objective:**

To elucidate the anti-inflammatory and anabolic effects of regulated expression of IL-4 in chondrocyte-scaffolds under *in vitro* inflammatory conditions.

**Methods:**

Mature articular chondrocytes from dogs (n = 3) were conditioned through transient transfection using pcDNA3.1.cIL-4 (constitutive) or pCOX-2.cIL-4 (cytokine-responsive) plasmids. Conditioned cells were seeded in alginate microspheres and rat-tail collagen type I matrix (CaReS®) to generate two types of tissue-engineered 3-dimensional scaffolds. Inflammatory arthritis was simulated in the packed chondrocytes through exogenous addition of recombinant canine (rc) IL-1β (100 ng/ml) plus rcTNFα (50 ng/ml) in culture media for 96 hours. Harvested cells and culture media were analyzed by various assays to monitor the anti-inflammatory and regenerative (anabolic) properties of cIL-4.

**Results:**

cIL-4 was expressed from COX-2 promoter exclusively on the addition of rcIL-1β and rcTNFα while its expression from CMV promoter was constitutive. The expressed cIL-4 downregulated the mRNA expression of IL-1β, TNFα, IL-6, iNOS and COX-2 in the cells and inhibited the production of NO and PGE_2_ in culture media. At the same time, it up-regulated the expression of IGF-1, IL-1ra, COL2a1 and aggrecan in conditioned chondrocytes in both scaffolds along with a diminished release of total collagen and sGAG into the culture media. An increased amount of cIL-4 protein was detected both in chondrocyte cell lysate and in concentrated culture media. Neutralizing anti-cIL-4 antibody assay confirmed that the anti-inflammatory and regenerative effects seen are exclusively driven by cIL-4. There was a restricted expression of IL-4 under COX-2 promoter possibly due to negative feedback loop while it was over-expressed under CMV promoter (undesirable). Furthermore, the anti-inflammatory /anabolic outcomes from both scaffolds were reproducible and the therapeutic effects of cIL-4 were both scaffold- and promoter-independent.

**Conclusions:**

Regulated expression of therapeutic candidate gene(s) coupled with suitable scaffold(s) could potentially serve as a useful tissue-engineering tool to devise future treatment strategies for osteoarthritis.

## Introduction

Osteoarthritis (OA) is the most common musculoskeletal disorder worldwide. It is the major cause of morbidity in developed nations and has enormous social and economic consequences. It is a slowly developing multifactorial disorder frequently associated with inflammation and progressive cartilage degeneration.

Progressive loss of cartilage in OA results from an imbalance of anabolic and catabolic metabolisms [1], [2] through a complex interaction of mechanical and biochemical factors [3], [4], [5]. Among the latter, a number of catabolic factors, including pro-inflammatory cytokines and proteases have been demonstrated to play major roles [1], [6], [7], [8].

Typically, repair in adult articular cartilage is very slow or even absent [9]. Cell based therapies using autologous mature chondrocytes or pre-chondrogenic stem cells in biodegradable polymeric tridimensional (3D) scaffolds when transplanted into focal lesions could regenerate hyaline-like cartilage [10], [11], [12]. However, pro-inflammatory mediators present in the joint could affect the transplanted chondrocytes, potentiating the need to suppress inflammation [13].

Although various biological factors have been independently identified as necessary for reducing inflammation or promoting regeneration, the most promising therapeutic agents are those that modulate the activities of the pro-inflammatory cytokines interleukin-1 beta (IL-1β) and tumor necrosis factor alpha (TNFα) which are thought to be important mediators that drive the pathophysiology of OA [8], [14], [15]. Several anti-inflammatory and anabolic agents have been tested that suppress the production of pro-inflammatory mediators [16], [17]. Among these IL-4 [18], IL-10 [19] and IL-13 [20] are of utmost significance in the context of OA.

We are interested in IL-4 because it has advantages over IL-10 or IL-13. As such, IL-4 compared to IL-10, is more potent inhibitor of IL-1β and only IL-4 (not IL-10) can induce the production of IL-1ra [21]. Further, IL-4 can antagonize the effects of TNF by inducing down-regulation and shedding of both forms of TNF receptors while IL-13 cannot produce such effects [22] and unlike IL-4, it does not appear to directly regulate the growth of Th2-type cells [23].

In addition, previous work using IL-4 under the control of constitutive [24] and responsive [25], [26] promoters revealed downregulation of various pro-inflammatory cytokines such as IL-1β, TNFα, IL-6 and enzymes involved in the production of inflammatory mediators such as inducible nitric oxide synthase (iNOS) and cyclooxygenase-2 (COX-2) and their end products nitric oxide (NO) and prostaglandin E_2_ (PGE_2_), respectively. According to Geurts and co-workers [25], IL-4 can protect cartilage erosion in collagen-induced arthritis and strongly reduces amounts of inflammatory cell influx.

Ideally, autologous chondrocyte transplantation (ACT) coupled with bioactive factors i.e. conditioning of cells with therapeutic transgenes may add regenerative and curing functions to the well-established repair function of conventional ACT [27]. This combination may be utilized to down-regulate inflammatory products and help restore the intrinsic biological function of the tissue. Thus, this approach would represent an interesting and unique modification of chondrocyte-seeded scaffolds that may substantiate future efforts to optimize ACT.

Our initial findings from alginate culture revealed a diminished expression of the inflammatory cytokines and other mediators in conditioned chondrocytes through cIL-4 production under both CMV (cytomegalovirus) and COX-2 promoters. These results were very promising and prompted us to test our hypothesis in rat-tail collagen type I CaReS® matrices, which are a suitable means to facilitate cell seeding of scaffolds for cartilage tissue engineering applications. Clinical trials of CaReS® matrices have shown promising results. It has been reported that patellofemoral transplantation of CaReS® matrices for two years showed a significant increase in International Knee Documentation Committee (IKDC) scores in 78.6% of patients [28]. It has been recently reported that CaReS® transplantation resulted in complete defects filling with superior quality repair tissue compared to Hyalograft-C, a hyaluronic-based scaffold at 2 years post-surgery [29].

We hypothesize that canine IL-4 (cIL-4) expression by chondrocytes could be induced in both scaffolds and anticipate that conditioned chondrocytes seeded in the 3D scaffolds will be able to abate inflammatory mediators and help regenerate cartilage simultaneously when implanted into the joint. As these scaffolds contain the cells previously conditioned with cytokine-responsive promoter that deliver the transgene only when the promoter will be turned on in the presence of IL-1β and TNFα, we are introducing a term for this kind of approach as Autologous Conditioned Cell Therapy (ACCT) for future *in vivo* experiments.

## Materials and Methods

### Isolation and culture of chondrocytes

All procedures in this study, with regard to sample collection from euthanized animals at Clinic of Small Animals, Freie Universität, Berlin, Germany, were conducted by authorized veterinarians in full agreement with the formal requirements as stated by the Animal Protection Office. However, for our *in vitro* study on such materials no approval was necessary because no direct contact with live animals was involved at all.

Adult articular cartilage tissues were harvested under sterile conditions from femoral condyles of five canine cadavers within 24 h post-euthanasia. Chondrocytes were isolated as previously described [30]. Briefly, cartilage was diced into 2–3 mm^2^ slices and digested in spinner flasks using a cocktail of enzymes consisting of 1 U/ml Collagenase P (Roche Diagnostics, Mannheim, Germany), 330 U/ml Collagenase CLS II (Biochrom, Berlin, Germany) and 30 U/ml Hyaluronidase (Roche Diagnostics, Mannheim, Germany) at 37°C for 16–18 h. The resulting suspension was filtered through a 100 µm cell strainer and chondrocytes were collected by centrifugation at 400 g for 20 min. Cells were then re-suspended in Dulbecco's Modified Eagle Medium (DMEM; Biochrom, Berlin, Germany) enriched with 10% heat inactivated fetal bovine serum (FBS; Biochrom, Berlin, Germany) and 100 U/ml penicillin and 100 mg/ml streptomycin (Pan Biotech, Aidenbach, Germany). Cells were counted and assessed for viability using trypan blue exclusion and plated in 75 cm^2^ culture flasks at 3×10^6^ cells*/*flask in the above-mentioned culture media and incubated at 37°C in 5% CO_2_. Based on good growth rate and phenotypic characteristics, cells from three donors (n = 3) at third sub-culture were used in this study.

### Preparation of DNA

cIL-4 was cloned into the pcDNA3.1 vector downstream of the constitutively expressing CMV promoter (pcDNA3.1.cIL-4) and downstream of the cytokine-responsive COX-2 promoter (pCOX-2.cIL-4; Accession No. EU249362; −1145 bp to +93 bp) by standard recombinant DNA technology [26], [31]. The activity of promoters and functioning of cIL-4 in monolayer culture were previously determined [26].

### Conditioning of chondrocytes

Chondrocytes at passage 3, were made conditioned through transient transfection using FuGENE 6 (Boehringer, Mannheim, Germany) [32] because this method yields approximately 50% transfection efficiency [26]. cIL-4 containing plasmids (pcDNA3.1-cIL-4, pCOX-2.cIL-4) were employed at 8 µg concentration (1 µg/µl) for individual transfection of 1×10^6^ cells in 10 cm cell culture dishes. A ratio of 3∶2 (FuGENE 6: DNA) was prepared in serum-free medium and added drop wise to the cells. Cells were maintained in DMEM plus 1% FBS and 1% penicillin/streptomycin at 37°C with 5% CO_2_.

### Encapsulation of chondrocytes in alginate microspheres

Chondrocytes were harvested 24 h post-transfection by trypsinization, washed twice with phosphate-buffered saline (PBS) and encapsulated in alginate microspheres [32]. In total, 10 microspheres (5 microspheres/ml) for each sample were placed in DMEM supplemented with 10% FBS and 1% penicillin/streptomycin and incubated as above for 24 h to allow equilibration of microspheres to culture.

### Generation of CaReS® matrices

Rat-tail collagen-based matrices (CaReS®) were generated 24 h post-transfection at Amedrix, Esslingen, Germany. Chondrocytes were harvested as above and resuspended in 2.5 ml of 2x gel neutralizing solution (GNS) for each matrix using 1.0×10^4^–1.5×10^4^ chondrocytes and mixed with 2.5 ml of collagen type I gel. The GNS/chondrocyte composite was cast for each gel, which was then allowed to polymerize at 37°C for 20 min to generate matrices. The matrices were placed in DMEM/F12 medium (Bioconcept, Allschwil, Switzerland) containing 10% FBS and 1% gentamicin (Biochrom, Berlin, Germany) for 24 h before stimulation.

### Simulation of inflammatory arthritis within scaffolds

After equilibration period was over, culture medium was aspirated and scaffolds were washed twice with PBS. Now, alginate beads were cultured in DMEM with 1% FBS while CaReS® matrices were cultured in DMEM/F12 with 1% FBS. Recombinant canine (rc) IL-1β and rcTNFα were used [33] to stimulate the inflammatory cascade in chondrocytes within both scaffolds at a concentration of 100 ng/ml and 50 ng/ml respectively for 96 h with essential controls.

### Neutralizing anti-cIL-4 antibody assay

To assess whether the anti-inflammatory and regulatory effects in conditioned chondrocytes are caused by the expression of cIL-4, neutralizing goat anti-canine-cIL-4 polyclonal antibody (AF754, R&D Systems, Wiesbaden-Nordenstadt, Germany) was added at a concentration of 5 µg/ml to the culture media of cells stimulated with recombinant canine pro-inflammatory cytokines for 96 h. Culture media were collected and subjected to the nitrite assay as described below.

### Retrieval of cells from scaffolds

Entrapped chondrocytes from alginate microspheres were re-isolated by digesting individual microspheres in 100 µl of 55 mM sodium citrate and 90 mM NaCl (pH 6.8) solution for 20 min at room temperature. Cells were released from CaReS® scaffolds by chopping the scaffolds to the size of a pinhead and subsequent incubation of the suspension with 1 ml (1.25 U) of Collagenase P with an equal volume of PBS. Samples were then incubated at 37°C for 20 min during which time the minced fractions were completely digested thereby releasing chondrocytes into the solution. The suspended chondrocytes from snapped alginate and CaReS® matrix solutions were pelleted and kept at −80°C until analyzed.

### RNA isolation and RT-PCR

Total RNA was extracted from chondrocytes using EURx GeneMATRIX universal RNA purification kit (Roboklon, Berlin, Germany) according to supplied protocol. The extracted RNA dissolved in DEPC-treated water was quantified by Nanodrop system (Peqlab, Erlangen, Germany) and treated with DNase I (Fermentas, St. Leon-Rot, Germany). One microgram of total RNA was used to synthesize first-strand cDNA using RevertAid Moloney murine leukemia virus reverse transcriptase and oligo(dT)_18_ (Fermentas, St. Leon-Rot, Germany) at 42°C for 60 min, according to manufacturer's protocol.

### Quantification of mRNA expression

mRNA expression of IL-1β, TNFα, IL-4, IL-6, iNOS, COX-2, insulin-like growth factor-1 (IGF-1), IL-1 receptor antagonist (IL-1ra), collagens (COL1a1, COL2a1) and aggrecan was quantified. Subsequently, the samples were subjected to quantitative real-time PCR (qRT-PCR) using an iCycler iQ-5 (Bio-Rad, Munich, Germany).

Reactions in triplicates were carried out in 20 µl reaction volume containing 10 µl of SensiMixPlus SYBR (Quantace, Berlin, Germany) with fluorescein and 3 mM MgCl_2_. Primers (Table-1) were used at 500 nM, the cDNA was added at a concentration of 500 ng (1 µg/µl). The gene for glyceraldehyde 3-phosphate dehydrogenase (G3PDH) acted as an endogenous reference for normalization of fluorescence thresholds (C_t_) values of target genes.

**Table 1:** Sequences and characteristics of the various oligonucleotide primers.

Gene symbol	S/A	Primer sequences (5′–3′)	Location	Size (bp)	NCBIaccession No.
IL-1β	S	AGTTGCAAGTCTCCCACCAG	149–169	177	DQ251036
	A	TATCCGCATCTGTTTTGCAG	325–345		
TNFα	S	TCATCTTCTCGAACCCCAAG	235–255	157	NM_001003244
	A	ACCCATCTGACGGCACTATC	391–411		
IL-6	S	GGCTACTGCTTTCCCTACCC	108–128	198	NM_001003301
	A	TTTTCTGCCAGTGCCTCTTT	305–325		
iNOS	S	GGAGGAGCAGCTACTGTTGG	1227–1246	178	AF068682
	A	GTCATGAGCAAAGGCACAGA	1385–1404		
COX-2	S	GCCTTACCCAGTTTGTGGAA	1239–1258	163	NM_001003354
	A	AGCCTAAAGCGTTTGCGATA	1382–1401		
IL-4	S	CTCACCTCCCAACTGATTCC	70–89	156	NM_001003159
	A	CTTGACAGTCAGCTCCATGC	206–225		
IGF-1	S	CAGCAGTCTTCCAACCCAAT	12–31	105	XM_848024
	A	CAAGCACAGTGCCAGGTAGA	98–117		
IL-1ra	S	GAAGAGACCTTGCAGGATGC	87–106	226	AF216526
	A	CTGGAGCCTGGTCTCATCTC	312–331		
COL1a1	S	GAACCTGGCAAACAAGGTC	3017–3035	150	NM_001003090
	A	AGGAGAACCATCTCGTCCA	3148–3166		
COL2a1	S	GAAACTCTGCCACCCTGAAT	3878–3897	160	NM_001006951
	A	GCTGCTCCACCAGTTCTTCT	4018–4037		
Aggrecan	S	CTATGAGGACGGCTTTCACC	573–592	194	U65989.2
	A	AGACCTCACCCTCCATCTCC	747–766		
G3PDH	S	TAT TGT CGC CAT CAA TGA CC	81–100	195	NM_01003142
	A	TAC TCA GCA CCA GCA TCA CC	261–275		

S = sense (forward); A = antisense (reverse); bp = base pairs.

### cIL-4 enzyme linked immunosorbent assay (ELISA)

The supernatant was collected from cells 96 h post-stimulation and cells were harvested by centrifugation and lysed using RIPA (radioimmunoprecipitation assay) buffer containing 1% Triton X-100, 1% deoxycholate, 0.1% SDS (sodium dodecyl sulfate), 0.15 M NaCl, 20 mM Tris, 10 mM EDTA (ethylenediaminetetraacetic acid), 10 mM iodoacetamide, 1 mM PMSF (phenylmethylsulfonyl fluoride) supplemented with protease inhibitors (10 µg/ml each of aprotinin, leupeptin, and pepstatin). Proteins present in the culture media were precipitated with the chloroform: methanol method [34]. Proteins present in lysates and in concentrated supernatants were quantified by the bicinchoninic acid method (Bio-Rad, Munich, Germany). To determine IL-4 protein, sandwich ELISA was performed on cell lysates and culture media from both matrices according to the previously established protocol [24]. Briefly, 96-well flat-bottomed plate (Nunc, Roskilde, Denmark*)* was coated with 200 µl/well of house-raised Rabbit polyclonal cIL-4 antibody (1∶1000) for 24 h at 4°C. After 3x washing with PBS, the plate was blocked using 200 µl of 1% bovine serum albumin in PBS for 2 h at room temperature followed by 3x washing with PBS. Undiluted samples and serial dilutions of standard rcIL-4 were dispensed in triplicates and incubated at room temperature for 90 min. Plate was rinsed 3x with PBS-Tween (0.1% Tween 20) followed by the addition of house-raised anti-mouse cIL-4 monoclonal antibody (1∶10). After 1 h incubation at room temperature, plate was washed 3x with PBS. Subsequently, 50 µl of biotin-labelled anti-mouse antibody (1∶2500) and streptavidin conjugated horseradish peroxidase (1∶4000) were delivered sequentially with washing steps in between. Lastly, 100 µl of substrate (12-oxo-phytodienoic acid in citrate buffer with 0.01% H_2_O_2_) was dispensed to each well of the plate and incubated in the dark for 30 min. The subsequent reaction was terminated with 1 M H_2_SO_4_ and plate was read at 492 nm at an ELISA reader.

### Collagen assay

The soluble collagen release in the culture media was determined by the Sircol collagen assay (Biocolor, Carrickfergus, UK). Absorbance was measured on a multi-well plate reader and compared to a plot of standards prepared from purified bovine collagen to determine total collagen contents [35].

### Sulphated glycosaminoglycan assay

Sulphated glycosaminoglycan (sGAG) released into the culture media was determined using a Blyscan glycosaminoglycan assay kit (Biocolor, Carrickfergus, UK). Absorbance was measured on a multi-well plate reader and compared to a plot of standards prepared from purified chondroitin-4-sulphate (derived from bovine trachea) [36].

### Assessment of NO

Concentration of nitrite, a stable product of NO was measured in samples taken from the culture supernatants by use of a colorimetric assay (Promega, Mannheim, Germany) based upon the Griess' reagent system [37].

### Assessment of PGE_2_

Aliquots of culture media were taken for PGE2 quantification using a PGE2 enzyme immunoassay kit (R&D Systems, Wiesbaden-Nordenstadt, Germany) [38].

### Statistical analysis

Statistical analysis was carried out with R (version 2.10.1) utilizing a two factorial ANOVA following a Tukey's Honestly Significant Difference (HSD) test for *post hoc* comparisons. The means of the three transfection groups and the means of two scaffold (Alginate/CaReS®) groups were tested for equality. The Tukey's HSD test was used to calculate critical values to determine significant differences of the means within the groups at p<0.05.

## Results

### Analysis of pro-inflammatory cytokines

**Figure 1:**
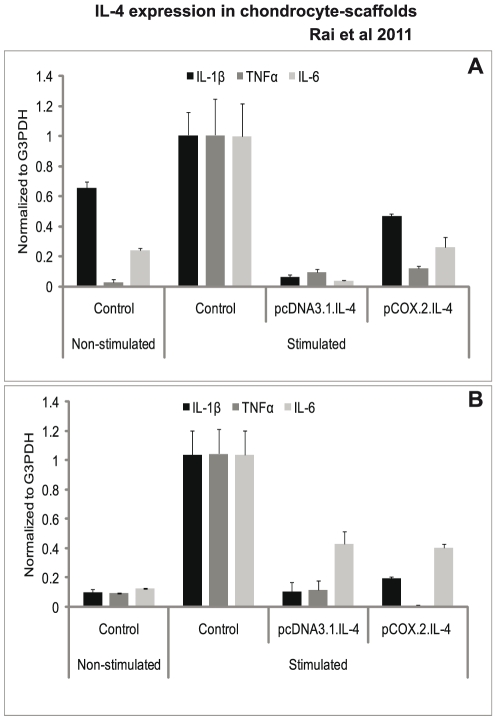
mRNA expression of proinflammatory cytokines. IL-4 transfected (pcDNA3.1.cIL-4 and pCOX-2.cIL-4) and non-transfected chondrocytes were seeded in alginate microspheres (A) and in CaReS® matrixes (B). Both scaffolds were stimulated with rcIL-1β (100 ng/ml) and rcTNFα (50 ng/ml) for 96 h. mRNA expression was quantified by qRT-PCR. The expression of IL-1, TNF and IL-6 was downregulated in IL-4 expressing scaffolds as compared to non-transfected controls on stimulation.

Our results show that the expression of cIL-4 driven by both CMV and COX-2 promoters in stimulated cells suppressed endogenous production of pro-inflammatory cytokines. As shown in Fig. 1A–1B, qRT-PCR analysis revealed that cIL-4 was able to suppress the expression of pro-inflammatory cytokines in both scaffolds and that cIL-4 expressed from both promoters gave similar inhibitory activity for endogenous expression of IL-1β, TNFα and IL-6.

### Analysis of destructive enzyme mediators

**Figure 2:**
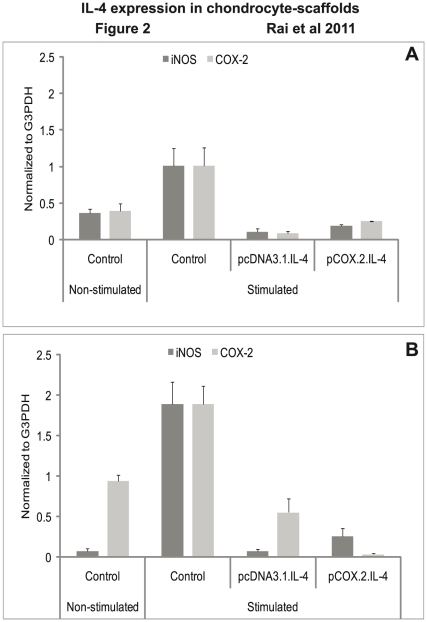
mRNA expression of enzyme mediators. IL-4 transfected (pcDNA3.1.cIL-4 and pCOX-2.cIL-4) and non-transfected chondrocytes were seeded in alginate microspheres (A) and in CaReS® matrixes (B). Both scaffolds were stimulated with rcIL-1β (100 ng/ml) and rcTNFα (50 ng/ml) for 96 h. mRNA expression was quantified by qRT-PCR. The expression of iNOS and COX-2 was downregulated in IL-4 expressing scaffolds as compared to non-transfected controls on stimulation.

As shown in Fig. 2A–2B, expression of iNOS and COX-2 was downregulated in conditioned chondrocytes. This indicates that cIL-4 expressed in the conditioned cells present in both scaffolds is capable of inhibiting the production of these (enzyme) mediators in both scaffolds. Control experiments showed that non-transfected chondrocytes expressed high levels of both iNOS and COX-2 only after exogenous stimulation with canine recombinant IL-1β and TNFα (results not shown).

### Analysis of regulatory mediators and matrix components

**Figure 3:**
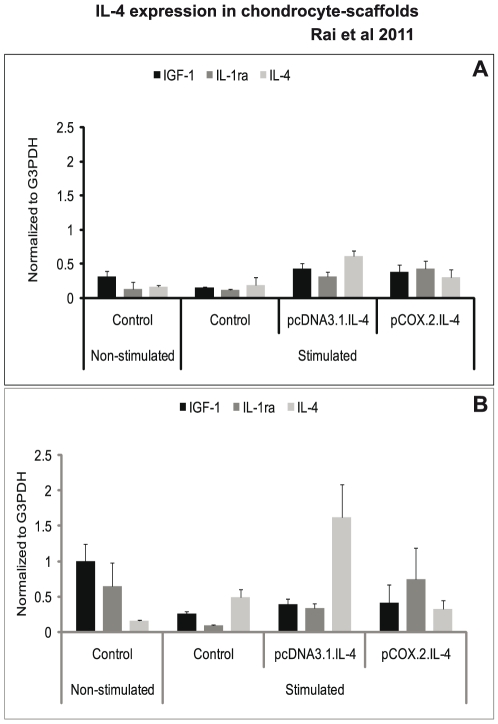
mRNA expression of regulatory mediators. IL-4 transfected (pcDNA3.1.cIL-4 and pCOX-2.cIL-4) and non-transfected chondrocytes were seeded in alginate microspheres (A) and in CaReS® matrixes (B). Both scaffolds were stimulated with rcIL-1β (100 ng/ml) and rcTNFα (50 ng/ml) for 96 h. mRNA expression was quantified by qRT-PCR. The expression of IGF-1, IL-1ra and IL-4 was up-regulated in IL-4 expressing scaffolds as compared to non-transfected controls on stimulation.

**Figure 4:**
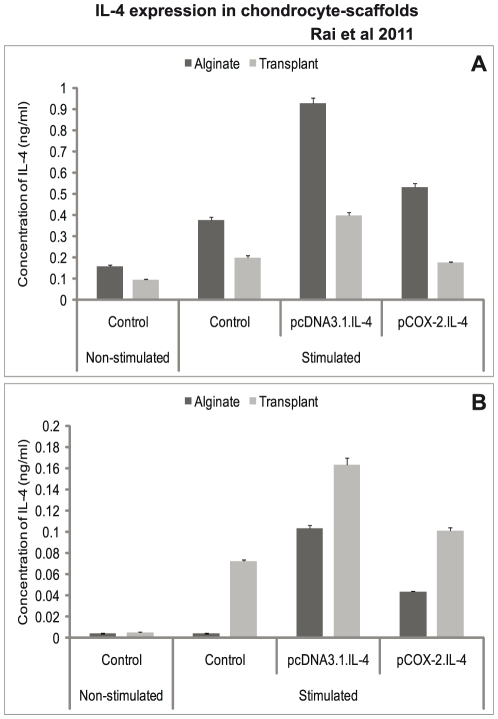
Detection of IL-4 protein. IL-4 transfected (pcDNA3.1.cIL-4 and pCOX-2.cIL-4) and non-transfected chondrocytes were seeded in alginate microspheres and in CaReS® matrixes. Both scaffolds were stimulated with rcIL-1β (100 ng/ml) and rcTNFα (50 ng/ml) for 96 h. Cell lysate and culture media were used to measure IL-4 production by sandwich ELISA. A lower IL-4 protein expression was found in cells conditioned with pCOX-2.cIL-4 as compared to those with pcDNA3.1.cIL-4 in lysates (A) and culture media (B). In both constructs, the expression was high as compared to non-transfected chondrocytes.

We observed that cIL-4 expressed from conditioned chondrocytes stimulated the production of IGF-1, IL-1ra, and IL-4 (Fig. 3A–3B) at higher levels as compared to the non-transfected control. Furthermore, expression yields for cIL-4 were apparently higher in cells conditioned with pcDNA3.1.cIL-4 than in those conditioned with pCOX-2.cIL-4. Irrespective of the type of scaffold, IGF-1 and IL-1ra were expressed at similar levels from both constructs. A sandwich ELISA revealed that the yield of cIL-4 protein was higher in lysates and in concentrated supernatants from the scaffolds that contained transfected cells (Fig. 4A–4B). Yet, basal levels of cIL-4 were also detectable in the stimulated, non-transfected cells.

**Figure 5:**
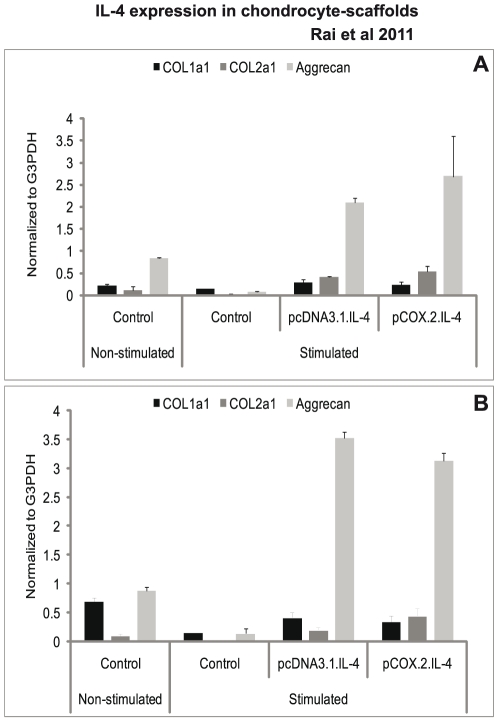
mRNA expression of collagens and aggrecan. IL-4 transfected (pcDNA3.1.cIL-4 and pCOX-2.cIL-4) and non-transfected chondrocytes were seeded in alginate microspheres (A) and in CaReS® matrixes (B). Both scaffolds were stimulated with rcIL-1β (100 ng/ml) and rcTNFα (50 ng/ml) for 96 h. mRNA expression was quantified by qRT-PCR. The expression of COL1a1 was downregulated whereas that of COL2a1 and aggrecan was up-regulated in IL-4 expressing scaffolds as compared to non-transfected controls on stimulation.

**Figure 6:**
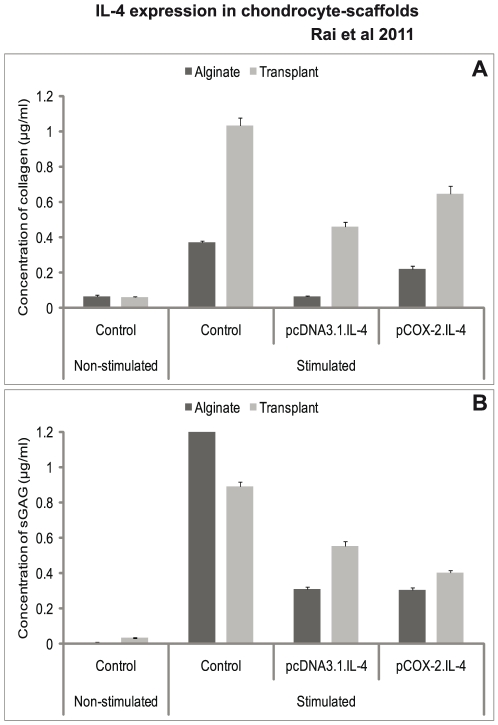
Release of total collagen and sGAG. IL-4 transfected (pcDNA3.1.cIL-4 and pCOX-2.cIL-4) and non-transfected chondrocytes were seeded in alginate microspheres and in CaReS® matrixes. Both scaffolds were stimulated with rcIL-1β (100 ng/ml) and rcTNFα (50 ng/ml) for 96 h. Supernatants were used to measure total collagen and sGAG release by respective assays. A diminished release of both collagen (A) and sGAG (B) was observed in the IL-4 expressing scaffolds as compared to non-transfected chondrocytes on stimulation.

The results in Fig. 5A–5B show that conditioned chondrocytes produced 10–20 fold more mRNA coding for COL2a1 than non-transfected cells. In contrast, the expression of COL1a1 was already quite high in non-transfected cell*s and became stimulated only by a factor of 3 to 4 in both scaffolds with cells transfected with cIL-4. We also show that the mRNA expression of aggrecan was significantly higher in IL-4-transfected chondrocytes in both scaffolds compared to non-transfected cells (Fig. 5A–5B). Furthermore, the release of total collagen and sGAG was also significantly lower in conditioned and stimulated cells as compared to that of the non-conditioned stimulated controls (Fig. 6A–6B).*

### Inhibition of NO production

**Figure 7:**
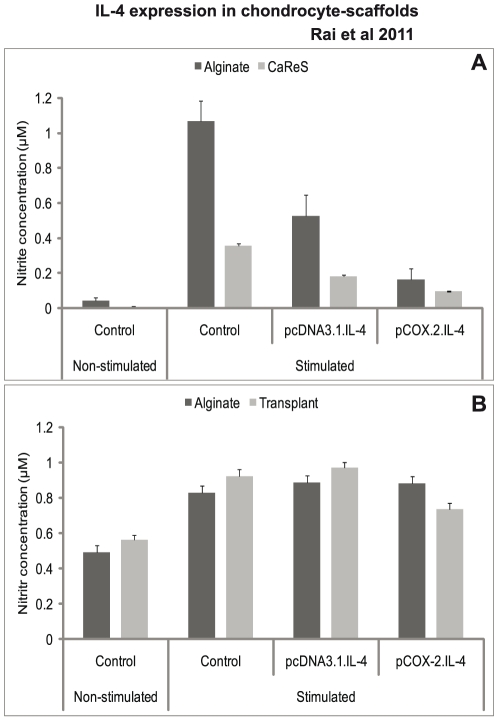
Determination of NO production. IL-4 transfected (pcDNA3.1.cIL-4 and pCOX-2.cIL-4) and non-transfected chondrocytes were seeded in alginate microspheres and in CaReS® matrixes. Both scaffolds were stimulated with rcIL-1β (100 ng/ml) and rcTNFα (50 ng/ml) for 96 h. Culture media were used to measure NO levels by using Griess' reagent system. There was a reduced NO production in IL-4 expressing scaffolds (A). However, there was no reduction in the NO production when the neutralizing anti-cIL-4 antibody was added in the culture media from both scaffolds (B).

As shown in Fig. 7A, nitrite levels are reduced in cells transfected with cIL-4. This is in line with the results presented in Fig. 2A–2B which suggest that iNOS is downregulated when cIL-4 is expressed.

### Neutralizing anti-cIL-4 antibody assay

As seen from the results shown in Fig. 7B the addition of antibody to the media prevented the downregulation of NO. These findings support our hypothesis that the anti-inflammatory and regenerative activities observed with both types of scaffolds containing conditioned cells may indeed be due to the expression of cIL-4.

### Inhibition of PGE_2_ production

**Figure 8:**
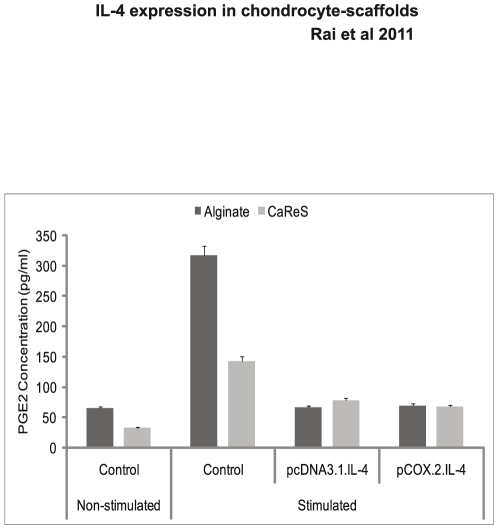
PGE_2_ determination. IL-4 transfected (pcDNA3.1.cIL-4 and pCOX-2.cIL-4) and non-transfected chondrocytes were seeded in alginate microspheres and in CaReS® matrixes. Both scaffolds were stimulated with rcIL-1β (100 ng/ml) and rcTNFα (50 ng/ml) for 96 h. Culture media were used to measure PGE_2_ levels by using PGE_2_ enzyme immunoassay kit. A diminished production of PGE_2_ was observed in IL-4 expressing scaffolds as compared to non-transfected.

Finally, we show that cIL-4 downregulated PGE_2_ production from the conditioned chondrocytes. The data shown in Fig. 8 are in line with those obtained for the levels of COX-2 expression (Fig. 2A–2B), all representing parameters associated with inflammatory arthritis.

## Discussion

Tissue engineering offers a plausible therapeutic approach to the repair of damaged cartilage [39], [40] through ACT [10], [11], [12]. Nevertheless, it has been shown previously that chondrocytes in scaffolds are susceptible to inflammatory mediators [33]. This scenario indirectly raises a question on the validity of ACT into cartilage lesions surrounded by progressive inflammation. In order to address this problem, we extended our previous work on the application of inducible cIL-4 expression in a chondrocyte-based model of inflammatory arthritis to 3D scaffolds. Our main objective to present this study is to examine whether cIL-4 produced within such 3D-biomaterials can downregulate inflammatory mediators and can recoup extracellular matrix synthesis.

We believe that this is the first study to validate the use of cytokine-therapy devoid of viral vectors in a 3D *in vitro* model of inflammatory arthritis. Inflammatory arthritis triggered by IL-1β and TNFα is widely accepted to be responsible for cartilage degradation and production of inflammatory mediator that further imply the central involvement of inflammation cascades in the early stages of OA [14], [15], [41]. Since chondrocytes were conditioned by transfection prior to generation of scaffolds, we coined a new term for this type of approach: ACCT (autologous conditioned cell therapy).

The data obtained prove that IL-4 is expressed at sufficient levels to effectively downregulate inflammatory mediators in both types of scaffolds. This indicates that both scaffolds containing conditioned chondrocytes allow unrestricted diffusion of cytokines in and out of the cells and through the matrix network into the surrounding culture medium. The results clearly indicate the anti-inflammatory activities of cIL-4. However, basal expression of some of the cytokines in non-stimulated and non-transfected control cells is in agreement with previous observations [42], [43].

While qRT-PCR results indicate mRNA levels, the estimation of catabolites such as NO and PGE_2_ in cell culture supernatants reflects the extent of inhibition of the inflammatory mediators. Moreover, since these mediators are end-products of the inflammation cascade, their suppression denotes the lower levels of pro-inflammatory cytokines. The diminished levels of NO (Fig. 7A) and PGE_2_ (Fig. 8) were observed, which emphasized the regulatory activity of cIL-4 on their respective catalyzing enzymes. NO also triggers chondrocyte apoptosis and initiates matrix metalloproteinases (MMPs) that degrade cartilage. As was observed previously, IL-4 has indeed inhibitory activity on MMPs (MMP-1, -3 and -13) [26].

While the anti-inflammatory activities of IL-4 are well known, restoration and/or enhancement of anabolic factors would form an ideal therapy in arthritis. One of the important characteristics of our approach is the ability of the pCOX-2.cIL-4 construct to deliver the therapeutic gene (in this case cIL-4) only upon stimulation with exogenous rcIL-1β and rcTNFα. As depicted in Figs. 3 and 4, less cIL-4 is expressed from the pCOX-2.cIL-4 construct as from the pcDNA3.1.cIL-4 construct. This is because the latter is expressed constitutively since driven from the CMV-promoter. This results in an over-production of cIL-4, which is not desirable because it has been reported that overexpression of IL-4 may result in severe joint inflammation that is characterized by synovial cell influx [44]. Thus, our approach is unique in controlling the expression of a therapeutic gene through the severity of inflammation as defined by the presence of pro-inflammatory cytokines. Therefore, we examined the applicability of a cytokine-responsive promoter for achieving efficacious IL-4 therapy under arthritic conditions, while minimizing IL-4-induced inflammatory arthritis under naive conditions [25].

At the same time, it is tempting to speculate that the expression of IL-4 under these promoters in both matrices is exclusively due to the presence of IL-4 cDNA in the vector constructs. In our preliminary studies in monolayer culture (Figure S1), we have determined that neither empty vectors (pcDNA3.1 and pCOX-2) nor transfection have any influence on the inhibition of above listed inflammatory mediators.

In line with our previous results, IL-4 has shown up-regulation of IGF-1 and IL-1ra. In addition, reports indicate that NO decreases IGF receptor tyrosine phosphorylation and hence decreases IGF-1 activity [45]. IGF-1 is known for its collagen matrix synthesis and for its anti-apoptotic functions. Stimulation of IL-1ra additionally has anti-inflammatory activities in that it antagonizes IL-1β. Thus, our observation that IL-4 up-regulates IL-1ra is in agreement with previous reports [46].

Another important observation in this study is the up-regulation of COL2a1, a matrix synthesizing protein specific for articular cartilage. Our results in Fig. 5 denote that IL-4 produced within a scaffold triggers the production of COL2a1, which is in line with a previous report for chondrocyte cultures [47]. Although COL1a1 also increases to some extent after constitutive expression of IL-4 from pcDNA.IL-4, the elevated ratios between COL2a1/COL1a1 in transfected chondrocytes indicate that IL-4 may contribute to stabilizing the re-differentiated state of chondrocytes present in the two scaffolds (Table 2). The biochemical assessment of the levels of collagen and sGAG, however, shows that IL-4 has an anabolic net effect on chondrocytes because the cIL-4-transfected chondrocytes show less breakdown of extracellular matrix components presumably due to the inhibited expression of MMPs [48], [49]. Although unlikely, the diminished release of sGAG in cell culture media may also be a consequence of decreased sGAG production or its retention in the scaffolds. To circumvent this issue and to substantiate our data, we measured the mRNA expression of aggrecan. Our results show that the expression of aggrecan is significantly increased in both scaffolds transfected with cIL-4 under both promoters (Fig. 5A–5B). We also made efforts to determine the expression of collagen type II at the protein level after 96 h of stimulation but it was not detectable by both immunocytochemistry and Western blot. We attribute this observation to the low numbers of cells present in the scaffolds, the detection limits of the assays and the relatively short incubation period. Currently, we present data at the mRNA level to monitor the expression of several marker genes for inflammatory arthritis and cartilage. Although it has been widely accepted that cytokine quantification in culture media is hard to determine, we attempted to quantify IL-4 protein in the cell lysate and in concentrated culture medium from transfected and non-transfected cells. It was shown, compared to non-transfected cells higher concentrations of IL-4 protein were detected in cell lysates and culture media from conditioned cells present in both types of 3D-cultures. However, the protein concentration was lower than that of monolayer cultures (not shown), most probably due to limited number of cells present in the scaffolds.

**Table 2:** COL2a1/COL1a1 ratio (differential index) in alginate and CaReS^®^ scaffolds.

	Transfection type		
Scaffold	Non-transfected	pcDNA3.1.cIL-4	pCOX.2.cIL-4
Alginate	0.157±0.008	1.410±0.007	2.250±0.121
CaReS®	0.224±0.112	0.472±0.022	1.290±0.061

The data obtained from mRNA expression of COL1a1 and COL2a1 was utilized to calculate the ratio between COL2a1 and COL1a1 (COL2a1/COL1a1) to show the differential index between two collagen types. It was shown that the differential index was significantly increased in cIL-4 transfected cells as compared to non-transfected controls overall showing high expression of COL2a1. All the samples were stimulated with rcIL-1β (100 ng/ml) and rcTNFα (50 ng/ml) for 96 h. Data is presented as mean ± S.D.

Using neutralizing anti-IL-4 antibody, we observed that addition of this antibody to the culture media prevented the downregulation of NO-synthesis by the conditioned cells present in the scaffolds. Apparently the secreted cIL-4 is captured by the antibody and hence is unable to act in a paracrine fashion which would otherwise lead to the interference with the inflammatory cascade eventually causing the observed drop in NO-production (compare Fig. 7A) [50]. These findings indicate that the anti-inflammatory and regenerative effects seen are directly related to IL-4 produced by the conditioned chondrocytes.

Overall, our study provides evidence that IL-4 produced by cells entrapped in a 3D scaffold such as alginate microspheres or a CaReS®-matrix can trigger both anabolic mediators and structural elements making this cytokine an ideal therapeutic candidate for use in 3D scaffolds utilized in transplantation.

This study directly compares both scaffolds and it appears that both alginate and CaReS® show reproducible results. We surmise that each of the scaffolds has its own advantage with alginate being easy to use and suitable for the repair of small cartilage defects but fragile during surgery. The other limitation of alginate may lie in potentially low rates of mass transport through the alginate matrix governed by diffusion. On the other hand, CaReS® matrices may be quite useful to cover larger cartilage lesions than alginate and are easy to handle as well. However, they would be more expensive. Future experiments using animal models as well as clinical trials will have to be performed in order to assess the practical applicability of ACCT. Ideally if the cytokine-responsive matrices described above do work in the patient as they do *in vitro*, a promising strategy for the treatment of OA may emerge in the future. While the data form *in vitro* experiments reported in this publication are promising in many respects, it is realized that future therapy trials will have to reveal, whether ACCT with scaffolds containing conditioned cells will satisfy expectations as an effective approach towards OA therapy.

## Supporting Information

Figure S1 Role of transfection and/or stimulation on the expression profile of selected markers of inflammatory arthritis. Chondrocytes in monolayer culture were treated just with transfection reagents (non-transfected) or mock transfected using empty pcDNA3.1 and pCOX-2 vectors under both stimulatory and non-stimulatory conditions. It was shown that there were only basal levels of expression of IL-1β, IL-6, iNOS and COX-2 in non-transfected, mock transfected and pcDNA.IL4 and pCOX-2-IL4 transfected cells without stimulation with rcIL-1β and rcTNFα. In contrast, on stimulation with rcIL-1β (100 ng/ml) and rcTNFα (50 ng/ml) for 96 h, only pcDNA.IL4 and pCOX-2-IL4 transfected cells were able to show a down-regulation of markers of inflammatory arthritis compared to non-transfected and mock transfected cell. This clearly indicate that the down-regulation of markers of inflammatory arthritis was exclusively due to IL-4 expression from the IL-4 containing constructs.
(EPS)
